# Nonsurgical periodontal therapy with/without 980 nm diode laser in patients after myocardial infarction: a randomized clinical trial

**DOI:** 10.1007/s10103-020-03136-6

**Published:** 2020-09-04

**Authors:** Renata Samulak, Mariusz Suwała, Elżbieta Dembowska

**Affiliations:** Department of Periodontology, Pomeranian Medical University in Szczecin, Szczecin, Poland

**Keywords:** Diode laser, Nonsurgical periodontal debridement, Periodontitis, myocardial infarction, Periodontal pathogens reduction

## Abstract

The purpose of this study was to evaluate the possible benefits (in terms of periodontal status improvement and periodontal bacteria count reduction) of using 980 nm diode laser in the treatment of periodontitis in patients after myocardial infarction. Thirty-six patients under 65 years of age (mean: 56.3 ± 7.9) with periodontitis, 6 weeks to 6 months after myocardial infarction, were recruited for the study. The control group (*n* = 18) received SRP (scaling, root planing and polishing) while the test group (*n* = 18) received SRP followed by laser therapy of the periodontal pockets with 980 nm diode laser, 1 W, continuous wave mode, 20 s per tooth side. Procedures were repeated twice at 5–7 day intervals. Microbiological and periodontal examination, including periodontal pocket depth (PPD), clinical attachment loss (CAL), bleeding on probing (BOP) and plaque control record (PCR), were performed before treatment, 2 weeks and 3 months after treatment. The study was registered on ClinicalTrials.gov with Identifier: NCT04145557, 29.10.2019 “retrospectively registered”. Additional use of laser resulted in a significant reduction in pockets with PPD ≥ 7 mm (*p* = 0.0151). The diode laser reduced total bacteria count (*p* = 0.0154) and delayed recolonisation during a 3-month observation period. A significant increase in the number of *Capnocytophaga gingivalis* was observed in the control group (*p* = 0.048). Additional use of the diode laser after SRP had no significant effect on BOP, CAL and PCR. Within the limitations of our study, we can conclude that 980 nm diode laser can be a useful tool in the treatment of periodontitis in patients after myocardial infarction.

## Introduction

Cardiovascular disease (CVD) including myocardial infarction (MI) caused 17.9 million deaths worldwide in 2016, representing 31% of all global deaths and it is the leading cause of mortality [1].

Current researches have shown that inflammation in the oral cavity, in particular periodontitis, affects the general state of health, including the development and course of atherosclerosis [2, 3]. Both periodontitis and CVD share similar risk factors and have an inflammatory origin [4–6]. Periodontitis is an infectious disease caused by periodontal bacteria resulting in inflammation, attachment loss, bone resorption and pocket formation [7, 8]. The prevalence of periodontal disease is high, but varies widely. Approximately > 90% of the world’s population have mild to advanced periodontitis [9] and there is 24% increased risk of coronary artery disease in periodontal patients after adjusting for important confounding factors [10].

The impact of periodontitis on the development of cardiovascular disease is complex. Initially, there is an increase in the level of pro-inflammatory mediators in the response to the presence of Gram-negative lipopolysaccharides, CRP (C-reactive protein), IL-1β, IL-6, TNF-α (tumour necrosis factor-α), fibrinogen and MMP-9 (metalloproteinase-9). They contribute to the destabilization of atherosclerotic plaque. Secondly, there is a cross-reaction of the patient’s antibodies with their own HS (heat shock) protein present in damaged vascular endothelium and atherosclerotic plaques. This leads to a progression of the disease. Cross-reactivity is triggered by the presence of oral bacteria, *Porphyromonas gingivalis* and *Tannerella forsythia*, whose thermal shock protein is 60% homologous with the heat shock protein found in mammals [11]. Thirdly, a direct bacterial mechanism (for example bacterial enzymes activity) leads to a progression in the disease. In atherosclerotical plaques bacterial DNA of *Tannerella forsythia*, *Porphyromonas gingivalis*, *Aggregatibacter actinomycetemcomitans* and *Prevotella intermedia* was found. Finally, the concept of “vascular endothelial activation” can explain the underlying mechanism of inflammatory induced atherosclerotic plaque formation. Ulcerated periodontal pocket epithelium enables penetration of lipopolysaccharides, bacterial outer membrane vesicles, fimbriae and other bacterial antigenic structures into the blood stream. Then they act as antigens and have an impact on local and systemic host response. This leads to an up-regulation of endothelial cell receptors followed by monocyte vascular wall adhesion. Monocytes migrate into the subendothelial space, absorb low density lipoprotein cholesterol (LDL) and become foam cells. After their apoptosis, lipids accumulate in the vessel wall and are covered by matrix, accompanied by smooth muscle cell proliferation which is induced by invasive periodontal pathogens. Enzymatic degradation of the extracellular matrix results in plaque rapture and exposition of prothrombotic components and subsequent thrombus formation, ultimately leading to blood vessel occlusion [12]. This results in the need for treatment of oral cavity diseases, as well as intensive promotion of periodontal disease prevention in patients with cardiovascular diseases [13, 14].

The basic nonsurgical treatment of periodontitis includes the mechanical debridement of the tooth surface from deposits, biofilm and toxins using machine and hand tools [15]. SRP (scaling, root planing and polishing) is effective in reducing inflammation, but does not completely eliminate it. Its effectiveness is limited in deep pockets, furcation areas and root depressions [16]. Diode lasers (DL) enable the effective removal of bacteria and toxins [17]. In addition to bactericidal and detoxification effects, a diode lasers can accelerate wound healing, facilitate collagen synthesis, accelerate angiogenesis and enable haemostasis [18–20]. Diode lasers are extremely effective in removing the epithelium with a thermal mechanism [21]. Another advantage of diode lasers is their small size and low cost [22]. In addition, deepithelialization with diode lasers requires less anaesthesia and is associated with less postoperative discomfort than compared with hand instruments [23]. Despite the potential benefits of laser therapy, the study results were not conclusive [24, 25]. The aim of this study was to evaluate the efficacy of the diode laser therapy as an adjunct to SRP in treatment of periodontitis in patients with MI. Most studies on the efficacy of a diode laser as an SRP support method focus on generally healthy patients. It is interesting to note whether such therapy is effective in patients after a myocardial infarction, whether it reduces both the need for surgical treatment and if it effectively eliminates bacteria associated with the development of CVD. The objective of this study was to compare the effects of SRP alone versus SRP + diode laser therapy with 980 nm laser, 1 W, continuous wave mode (CW), repeated 3 times in 2 weeks in patients after MI who also deal with periodontitis by means of clinical periodontal parameters (PPD, CAL, BOP) and microbial reduction (using PCR method) in 3-month observation period.

## Material and methods

**Fig. 1:**
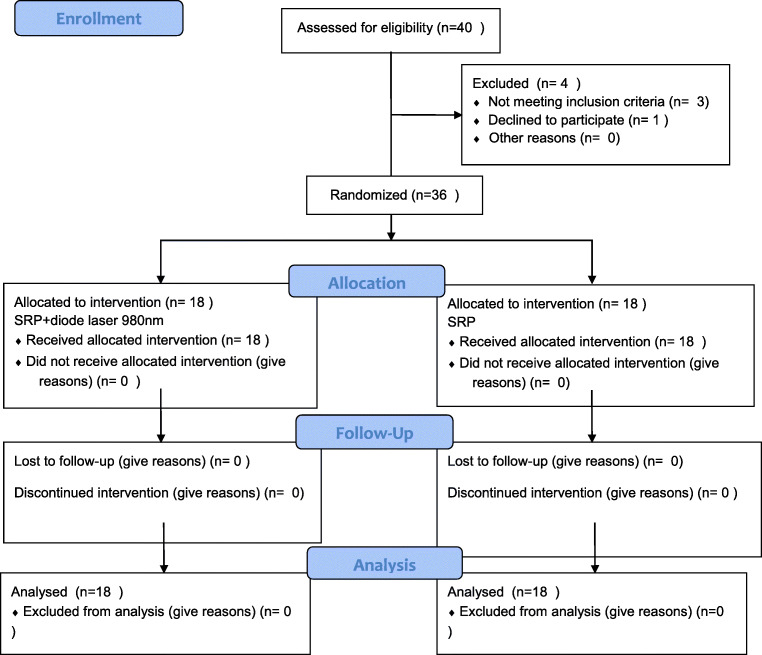
CONSORT 2010 flow diagram of the study

This study was a randomized and controlled 3-month clinical trial using a parallel design. Initially, 40 patients were invited to join the study. They were referred to the Department of Periodontology at the Pomeranian Medical University in Szczecin, Poland, for periodontal treatment between June 2019 and October 2019. These patients had been previously hospitalised at the Department of Cardiology due to myocardial infarction. The inclusion and exclusion criteria are in Table 1. Unfortunately, three patients did not meet the criteria, and one refused to participate (Fig. 1). Finally, the study covered 36 patients under 65 years of age (mean: 56.3 ± 7.9). Seven of them were female (19.4%) (Table 2). Written informed consent was obtained from all subjects. Patients diagnosed with periodontitis were randomly assigned into two groups. The control group (*n* = 18) received SRP and the test group (*n* = 18) received SRP followed by diode laser therapy of the periodontal pockets.

**Table 1:** Exclusion and inclusion criteria

Inclusion criteria	1. Signed informed consent 2. Myocardial infarction treated with primary coronary angioplasty in the last 6 weeks to 6 months 3. Age < 65 years 4. Periodontitis diagnosed according to Page criterion - • ≥ 2 tooth surfaces on interproximal spaces with a loss of CAL ≥ 4 mm (not for the same tooth) • ≥ 2 tooth surface in the interproximal space with PD ≥ 4 mm • Positive bleeding on probing test (BOP)
Exclusion criteria	1. Acute inflammation of the airways or urinary tract 2. Neoplasmas 3. Rheumatic disease 4. Autoimmune diseases 5. Chronic liver disease 6. Chronic kidney failure stage 4 or 5 7. History of stroke or transient ischemic attack (TIA) 8. Lack of consent for participation in the study 9. Antibiotic therapy in the last 12 months 10. Periodontal treatment in the last 6 months 11. Participation in other studies

**Table 2:** Demographic and periodontal characteristic of studied population at baseline

Parameters	Studied population
*n*	36
Age (years)	56.3 (± 7.9)
Gender	
Male	29 (80.6%)
Female	7 (19.4%)
BMI (body mass index)	27.64 (± 4.02)
Smoking	
No	30 (83.33%)
< 10/day	6 (16.67%)
≥ 10/day	0 (0%)
Myocardial infarction localization	
STEMI (ST elevation myocardial infarction)	31 (86.11%)
• Anterior	17 (47.22%)
• Lateral	3 (8.33%)
• Posterior	9 (25%)
NSTEMI (no ST elevation myocardial infarction)	5 (13.89%)
Coronary artery involvement	
LM (left main coronary artery)	14 (38.89%)
LAD (left anterior descending artery)	27 (75%)
Cx (circumflex artery)	21 (58.33%)
RCA (right coronary artery)	19 (52.78%)
Systemic diseases	
Hypertension	33 (91.67%)
Diabetes mellitus	7 (19.44%)
Dyslipidemia	29 (80.56%)
Osteoporosis	0 (0%)
Drugs	
ASA (acetylsalicylic acid)	36 (100%)
Clopidrogel	32 (88.89%)
Statins	27 (75%)
β-Blockers	33 (91.67%)
Angiotensin-converting enzyme (ACE) inhibitors	27 (75%)
Diuretics	10 (27.78%)
Periodontal status	
Number of teeth per patient	18.8 (± 5.4)
PPD (mm)	3.41 (± 1.03)
CAL(mm)	3.02 (± 2.62)
BOP (%)	56% (± 35%)
PCR	0.46 (± 0.31)

Periodontal examination was performed before treatment, as well as 2 weeks and 3 months after treatment with a PCP15 probe calibrated at intervals 1-2-3-4-5-6-7-8-9-10-11-12-13-14-15 mm (Hu-Friedy, USA). Periodontal pocket depth (PPD), clinical attachment loss (CAL) bleeding on probing (BOP) and plaque control record (PCR) measurements were obtained.

### Periodontal pocket depth

PPD is measured in millimetres from the free gingival margin to the base of the probable pocket using a periodontal probe at six sites per tooth (mesiobuccal, midbuccal, distobuccal, mesiolingual, midlingual and distolingual).

### Clinical loss of the attachment

CAL is defined as the distance from the cementoenamel junction to the base of the probable pocket at six sites per tooth (mesiobuccal, midbuccal, distobuccal, mesiolingual, midlingual and distolingual).

### Bleeding on probing according to Ainamo and Bay

The bleeding index during probing evaluates bleeding from probed sites after 10–30 s. It is expressed as a percentage of the places where the bleeding occurred in all of the examined sites. This indicator is examined at four sites around each tooth: mesiobucal, midbuccal, distobuccal and lingual.

### The plaque control record by O’Leary, Drake and Naylor

A dichotomous indicator that assesses the presence of dental plaque on four tooth surfaces—distal, buccal, mesial and palatal/lingual. It is expressed as a percentage of places where bacterial plaque is located on all of the examined sites.

#### Microbiological examination

A microbiological examination (via Real-PCR method) was performed using commercial standard sets PET-MIP plus ® (MIP Pharma, Germany). Samples were taken from the patient’s deepest periodontal pocket to assess the general number of bacteria (TBC, total bacteria count) and the number of periopatogenic bacteria: *Aggregatibacter actinomycetemcomitans* (*A. a.*), *Porphyromonas gingivalis* (*P. g.*), *Treponema denticola* (*T. d.*), *Tannerella forsythia* (*T. f.*), *Micromonas micros* (*M. m.*), *Prevotella intermedia* (*P. i.*), *Fusobacterium nucletum* (*F. n.*), *Eubacterium nodatum* (*E. n.*), *Capnocytophaga gingivalis* (*C. g.*). After the examined tooth was isolated from exposure to saliva, a sterile paper point was placed inside the pocket for 30 s following transfer to transport containers included in the PET-MIP deluxe ® kits and was then sent to the MIP-Pharma laboratory in St. Ingbert, Germany. Real time PCR analysis uses rapid duplication of selected DNA or RNA fragments enabling quantitative material evaluation even with low genetic material content. According to information from the manufacturer, PET-MIP plus ® test detects bacteria from 10^3^ threshold.

#### Time schedule

- Preeliminatory examination: 1 week before the study all interested, patients were informed regarding the study. Informed consent was signed. Then, detailed medical history interviews took place to establish exclusion and inclusion criteria.
- First examination E1: Periodontal examinations including PPD, CAL, BOP, PCR and microbiological samples were taken, following assignment into test and control groups. Periodontal treatment, in accordance with the study plan, was performed (SRP + diode laser in the test group and SRP in the control group).
- Second examination E2—2 weeks after E1. PPD, CAL, BOP and PCR were measured and microbiological samples were taken.
- Third examination E3—3 months after E1. PPD, CAL, BOP and PCR were measured and microbiological samples were taken.

#### Clinical procedure

In the control and study group, supra and subgingival scaling with ultrasonic tip PIEZON 250® (EMS, Switzerland) and root smoothing with Gracey currets (Hu-Friedy, USA) were performed. Tooth crowns were polished with Cleanic™ paste (Kerr, USA) and brushed with a handpiece at 2500–3000 rpm. Periodontal pockets were rinsed with saline. Procedures were repeated twice at 5–7 day intervals. In addition, pocket laser therapy with a 980 nm diode laser (Smart3M, Lasotronix, Poland) was carried out in the test group at each of the three treatment visits. After scaling, root planning and pocket flushing with saline, protective glasses were worn by the patient and the doctor. Laser therapy was carried out with an optic fibre with 200 μm diameter in continuous wave mode (CW) with 1 W power output. The power density of the fibre tip was 3184.7 W/cm^2^ and fluency 640 J/cm^2^. Total energy per tooth side was 20 J. This was then introduced into the periodontal pockets from the last tooth in the upper right quadrant of the oral cavity. The fibre tip was inserted into the periodontal pocket base in parallel alignment with the root surface, the device was activated and the fibre was slowly (1 mm/s) moved from apical to coronal in a sweeping motion during the laser light emission, not exceeding 40 s per tooth or 20 s per tooth surface. The fibre tip was cleaved before each irradiation to maintain its physical properties. Any debris accumulated or carbonisation of the tip was removed. After each laser disinfection, pockets were rinsed with saline. Oral hygiene instructions were given to each patient (roll technique, dental floss or interdental brush according to periodontal status). The treatment was performed by an experienced clinician (RS). All clinical measurements were performed by an experienced investigator (MS) who was blind of the division of subjects during the whole study period. Calibration of the individual examining periodontal parameters took place before the start of the study and consisted of examining the tooth pocket depth twice in 48 h intervals in patients with advanced periodontitis, who were not participating in the study. Calibration was considered successful if the measurements were consistent up to 1 mm in 90% of the tooth surfaces evaluated. Patients were not aware of which group they were assigned to until interventions were performed. The statistician was blind of the division of subjects during the whole study period.

#### Statistical analysis

PPD was set as the primary outcome and used to estimate the sample size. If a PPD difference was to be detected in the change between methods of 1 mm at α = 0.05 with 82% power, the appropriate number of participants per group were 15 patients. To ensure there were enough patients, 18 individuals were recruited per group. All continuous variables were checked for distribution normality using the Kolmogorov-Smirnov test. Statistical differences between the two groups were checked using the Student or Mann-Whitney *t* test. A variance analysis test (ANOVA) or Kruskal -Wallis test was used for many groups. The differences that were statistically significant in all tests were those in which there was a probability of *p* < 0.05. The power of the tests was set at 0.80. Statistical analyses were performed using the STATA11 statistical program (2009) license number 3010532736.

## Results

The treatment was uneventful in all cases and no adverse effects were noted. There was a statistically significant reduction in the BOP ratio and PCR in both groups after 3 months post-treatment but without statistically significant differences between the groups (Table 3).

**Table 3:** BOP, PCR, PPD and CAL in test and control group at baseline (E1), 2 weeks (E2) and 3 months after treatment (E3)

Parameter	examination	Test group *n* = 18				Control group *n* = 18				
		MD	SD	Min	Max	MD	SD	Min	Max	P
BOP (%)	E1	61	37	0.00	100	51	33	3	100	0.4633
	E2	30	25	0.00	84	16	11	0.00	41	0.1498
	E3	12	14	0.00	50	19	14	0.00	58	0.0640
	p	0.0002*				0.0006*				
PCR (%)	E1	43	32	3	100	50	31	6	100	0.3666
	E2	18	11	3	45	16	12	0.00	41	0.4107
	E3	12	12	0.00	40	21	15	0.00	50	0.0614
	p	0.0006*				0.0006*				
PPD (mm)	E1	3.48	0.89	2.37	5.34	3.34	1.17	2.11	6.36	0.3672
	E2	2.88	0.76	1.72	4.69	2.67	0.66	1.98	4.43	0.3757
	E3	2.79	0.61	1.60	3.70	2.67	0.54	2.07	4.31	0.2418
	p	0.0530				0.0725				
CAL (mm)	E1	2.77	2.59	0.12	8.54	3.27	2.70	0.26	9.19	0.6127
	E2	2.81	2.60	0.10	8.55	3.30	2.73	0.26	9.19	0.4668
	E2	2.78	2.57	0.10	8.55	3.21	2.89	0.00	9.19	0.6578
	p	0.9491				0.9714				

*p < 0.05 is statistically significant

**Fig. 2:**
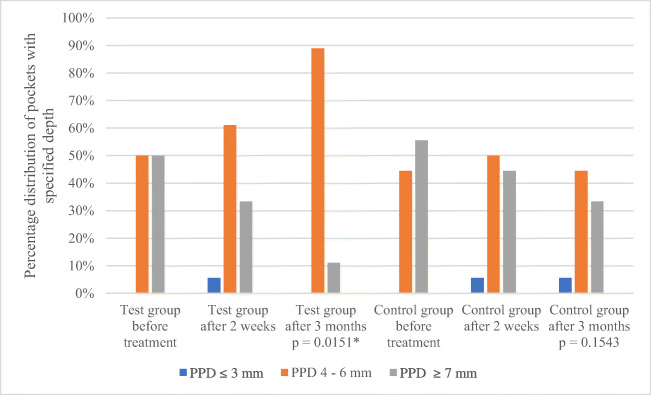
PPD in test and control group at baseline, 2 weeks and 3 months after treatment. There was a statistically significant reduction of percentage of pockets deeper than 7 mm in the test group. *p < 0.05 is statistically significant

The average PPD at the beginning of treatment (E1) in the test group was 3.48 mm (± 0.89 mm) and 3.34 mm (± 1.17 mm) in the control group. There were no statistically significant differences between groups (*p* = 0.3662). After completion of the observation (E3), PPD in the test and control group was 2.79 mm (± 0.61 mm), *p* = 0.053, and 2.67 mm (± 0.54 mm), *p* = 0.0725, respectively without statistically significant differences between groups (*p* = 0.2418) (Table 4). In the test group, the reduction of PPD was close to the level of statistical significance. Further analysis, dividing the pockets into three categories: shallow (≤ 3 mm), medium deep (4–6 mm) and deep (≥ 7 mm) showed that that there were no shallow pockets at the baseline, in both groups. In the test group, both medium and deep pockets accounted for 50% of each. In the control group, medium and deep pockets constituted 44.44% and 55.56%, respectively. After 3 months of treatment, the distribution of shallow, medium and deep pockets in the test group was 0%, 88.89%, 11.11%, respectively, and this was a statistically significant difference (*p* = 0.0151). In the control group, on the other hand, pockets ≤ 3 mm, 4–6 mm, ≥ 7 mm accounted for 5.56%, 61.1% and 33.33%, respectively, without any statistically significant difference (*p* = 0.1543). This means that in the test group, the majority of ≥ 7 mm pockets were reduced and joined the 4–6 mm pocket group, hence the overall average pocket depth did not significantly change (Table 4, Fig. 2).

**Table 4:** Percentage of patients with periodontal pockets with PPD ≤ 3 mm, 4–6 mm, ≥ 7 mm and CAL ≤ 2 mm, 3–4 mm, ≥ 5 mm at baseline (E1), 2 weeks (E2) and 3 months after treatment (E3)

	Test group *n* = 18			Control group *n* = 18		
PPD	E1	E2	E3	E1	E2	E3
≤ 3 mm	0%	5.56%	0%	0%	5.56%	5.56%
4–6 mm	50%	61.11%	88.89%	44.44%	50%	61.11%
≥ 7 mm	50%	33.33%	11.11%	55.56%	44.44%	33.33%
	Chi^2^ Pearson 8.52; df = 4; *p* = 0.07430 *R* Spearman rank 0.33; *t* = 2.513; *p* = 0.01510*			Chi^2^ Pearson 2.50; df = 4; *p* = 0.64464 R Spearman rank 0.2; *t* = 1.449; *p* = 0.1543		
CAL	E1	E2	E3	E1	E2	E3
≤ 2 mm	0%	0%	0%	0%	0%	0%
3–4 mm	11.11%	5.56%	0%	0%	0%	5.56%
≥ 5 mm	88.89%	94.44%	100%	100%	100%	94.44%
	Chi^2^ Pearson 2.12; df = 4; *p* = 0.71413 R Spearman rank 0.20; *t* = 1.4569; *p* = 0.15117			Chi^2^ Pearson 2.04; df = 4; *p* = 0.77882 R Spearman rank 0.17; *t* = 1.231; *p* = 0.22398		

*p < 0.05 is statistically significant

**Fig. 3:**
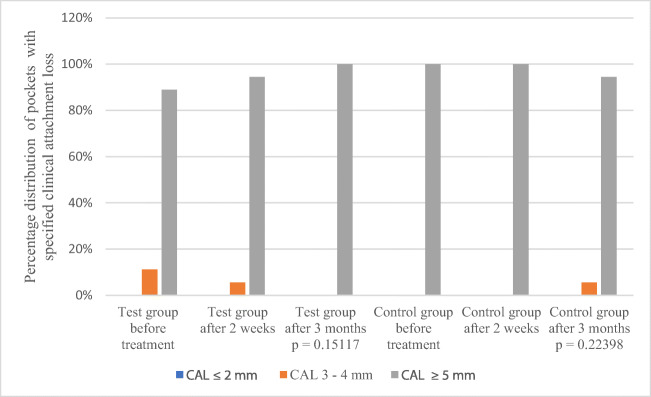
CAL in test and control group at baseline, 2 weeks and 3 months after treatment. There were no statistically significant differences between the test and control group before and after treatment in CAL. *p* < 0.05 is statistically significant

The average CAL at the beginning of the observation period (E1) in the test group was 2.77 mm (± 2.59 mm) and in the control group 3.27 mm (± .70 mm) without statistically significant differences between groups (*p* = 0.6127). After treatment (E3), CAL in the test group was 2.78 mm (± 2.57 mm) *p* = 0.949 and in the control group 3.21 mm (± 2.89 mm) *p* = 0.9714, again without statistically significant differences between the groups (*p* = 0.6578) (Table 3). The division of CAL into three categories (≤ 2 mm, 3–4 mm, ≥ 5 mm) did not reveal statistically significant differences between the groups, but in the test group, the increase in the percentage of patients with CAL ≥ 5 mm was clinically noticeable (from 88.89% to 100%) (Table 4, Fig.3).

**Fig. 4:**
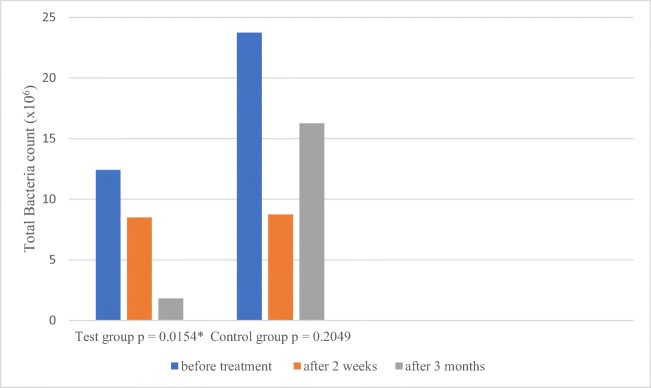
Total bacteria count in test and control group at baseline, 2 weeks and 3 months after treatment. There was statistically significant reduction of total bacteria count in test group after 3 months after treatment. *p < 0.05 is statistically significant

There were no statistically significant differences between groups prior to treatment initiation (*p* = 0.6350) in terms of total bacterial count—in the test and control group 12.42 × 10^6^ (± 19.82 × 10^6^) and 23.75 × 10^6^ (± 39.00 × 10^6^), respectively. In the second examination (E2), the number was 8.48 × 10^6^ (± 14.55 × 10^6^) and 8.73 × 10^6^ (± 16.78 × 10^6^), respectively. In the third examination, this number was further reduced to 1.80 × 10^6^ (± 2.70 × 10^6^) in the test group and this was a statistically significant reduction (*p* = 0.0154). In E3, the control group showed an increase compared with E2 up to 16.26 × 10^6^ (± 40.94 × 10^6^), causing the total reduction in bacterial counts between the initial and third examination without statistical significance (*p* = 0.2049) (Table 5, Fig. 4). In both groups, the number of *Porphyromonas gingivalis, Treponema denticola*, *Tannerella forsythia* and *Micromonas micros* saw a statistically significant decrease, without statistically significant differences between the groups. A statistically significant (*p* = 0.0444) reduction in the number of *Eubacterium nodatum* bacteria from 0.04 × 10^3^ (± 0.09 × 10^3^) to 0.00 × 10^3^ (± 0.00 × 10^3^) was observed in the test group. In the control group, this reduction was not statistically significant (*p* = 0.4019) and *Capnocytophaga gingivalis* increased significantly from 26.79 × 10^3^ (± 52.09 × 10^3^) to 139.11 × 10^3^ (± 246.83 × 10^3^) *p* = 0.0484. While in the test group the bacterium was reduced from 13.43 × 10^3^ (± 23.98 × 10^3^) to 4.44 × 10^3^(± 7.55 × 10^3^) *p* = 0.2621. This difference was statistically significant between groups (*p* = 0.0488) (Table 5, Fig. 4).

**Table 5:** Bacteria count in test and control group at baseline (E1), 2 weeks (E2) and 3 months after treatment (E3)

Bacteria	Examination	Test group *n* = 18				Control group *n* = 18				
		MD	SD	Min	Max	MD	SD	Min	Max	*p*
Total bacteria count (10^6^)	E1	12.42	19.82	0.09	73.00	23.75	39.00	0.02	150.00	0.6350
	E2	8.48	14.55	0.00	49.36	8.73	16.78	0.01	69.00	0.8494
	E3	1.80	2.70	0.02	8.90	16.26	40.94	0.01	170.00	0.2680
	p	0.0154*				0.2049				
*A. a.* (10^3^)	E1	0.19	0.57	0.00	2.10	0.00	0.00	0.00	0.00	0.2583
	E2	0.09	0.40	0.00	1.70	0.00	0.00	0.00	0.00	0.3173
	E3	0.01	0.06	0.00	0.26	0.00	0.00	0.00	0.00	0.9682
	p	0.4217				0.6005				
*P. g.* (10^3^)	E1	52.67	85.18	0.00	270.00	79.10	196.30	0.00	800.00	0.8554
	E2	9.00	32.85	0.00	140.00	4.63	17.22	0.00	73.00	0.2794
	E3	11.61	47.05	0.00	200.00	3.96	14.15	0.00	60.00	0.7393
	p	0.0334*				0.0217*				
*T. d.* (10^3^)	E1	21.87	62.52	0.00	270.00	38.37	60.25	0.00	200.00	0.9747
	E2	23.33	91.63	0.00	390.00	2.05	5.24	0.00	20.00	0.6774
	E3	1.82	3.59	0.00	11.00	3.15	12.94	0.00	55.00	0.7343
	p	0.0016*				0.005*				
*T. f.* (10^3^)	E1	31.93	57.88	0.00	200.00	22.62	35.02	0.00	130.00	0.7872
	E2	38.34	157.66	0.00	670.00	0.32	0.90	0.00	3.80	0.4153
	E3	2.91	8.57	0.00	35.00	1.60	6.59	0.00	28.00	0.1871
	p	0.0028*				0.0001*				
*P. i.* (10^3^)	E1	26.66	60.94	0.00	250.00	103.69	220.06	0.00	900.00	0.3043
	E2	19.69	66.78	0.00	280.00	46.76	124.43	0.00	440.00	0.6107
	E3	4.33	15.20	0.00	64.00	0.11	0.45	0.00	1.90	0.1555
	p	0.1715				0.0013*				
*M.m.* (10^3^)	E1	60.26	177.75	0.00	740.00	10.86	15.75	0.00	49.00	0.9621
	E2	4.68	10.60	0.00	35.00	4.11	7.73	0.00	30.00	0.9746
	E3	1.87	3.20	0.00	9.20	0.28	0.42	0.00	1.40	0.5251
	p	0.0349*				0.0006*				
*F. n.* (10^3^)	E1	25.25	65.44	0.00	270.00	5.83	11.84	0.00	39.00	0.7099
	E2	2.62	5.22	0.00	20.00	6.75	13.63	0.00	54.00	0.5263
	E3	2.55	9.86	0.00	42.00	8.77	30.43	0.00	130.00	0.2858
	p	0.1847				0.8051				
*E. n.* (10^3^)	E1	0.04	0.09	0.00	0.32	0.22	0.65	0.00	2.40	0.9417
	E2	0.00	0.00	0.00	0.00	0.01	0.04	0.00	0.15	0.3173
	E3	0.00	0.00	0.00	0.00	0.05	0.23	0.00	0.96	0.3173
	p	0.0444*				0.4019				
*C. g.* (10^3^)	E1	13.43	23.98	0.00	100.00	26.79	52.09	0.00	1§0.00	0.5258
	E2	74.47	281.48	0.00	1200.00	18.48	36.82	0.00	130.00	0.8483
	E3	4.44	7.55	0.00	23.00	139.11	246.83	0.00	810.00	0.0488*
	p	0.2621				0.4841				

*p < 0.05 is statistically significant

## Discussion

The treatment of periodontitis with SRP has a substantial clinical efficacy; however, it does not always eradicate the pathogenic bacteria species due to their location within the periodontal tissue or other areas inaccessible by periodontal instruments [15]. Hence, there is a need for additional antibacterial protocols to eliminate microbes. In the era of increasing resistance of microorganisms to antibiotics and the high risk of antibiotic therapy side effects, additional laser therapy could be beneficial [25]. However, the most important meta-analyses and systematic reviews have not provided clear conclusions [19, 25–27]. Sgolastra et al. [19] in a cautious meta-analysis based on 4 studies concluded that diode laser as an adjunct therapy to conventional nonsurgical periodontal treatment did not provide additional clinical benefits. However, meta-analysis combined studies from different phases of treatment (initial and maintenance therapy) and different types of laser therapy—LLLT (Low Level Laser Therapy) therapy and high-power laser therapy. Slot et al. [25] in a systematic review and meta-analysis based on 9 studies [22, 29–36] questioned the legitimacy of using a diode laser as an additional method supporting SRP. The adjunctive use of DL is judged to be moderate for changes in PPD and CAL. In terms of bleeding scores, better results were obtained in a laser group, but any clinical relevance of this difference remains questionable. Quadri et al. [28] in systematic review based on 10 trials [22, 29–37] concluded that diode lasers with SRP are more effective in patients with periodontitis and pockets probing depths ≤ 5 mm than when SRP is used alone. They noted that the lack of efficiency and even worse results in laser groups occur in studies where 810 nm lasers were used [22, 31] (high absorption in haemoglobin, possible carbonisation within irradiated tissues) and where continuous wave mode was used [22, 31] (heat accumulation, carbonisation possibility). Another cause for the failures is the use of insufficient power (0.8 W) [32]—which seemed ineffective in the elimination of epithelium of periodontal pockets and bacteria.

In our study we achieved a statistically significant reduction in BOP and PI in both groups, but without statistically significant differences between treatment methods. Close results were obtained in several studies [30, 33, 34, 38] using a 980 nm diode laser as an additional method after SRP. In some of these studies [33] single laser irradiation was performed, while in others the procedure was repeated several times. Caruso et al. [34] carried out a laser irradiation for 30 s twice in one visit, Yadwad et al. [38] also treated single pocket twice for 30 s with a 60-s break on one visit and repeated the procedure after 1 week. Dukić et al. [30] performed a single 20-s irradiation of one treated tooth in one visit and repeated this procedure after 3 and 7 days. This laser therapy scheme was the closest to our own, as we used 20-s irradiation on the tooth side and repeated it twice within 2 weeks after first appointment.

These studies were consistent in the case of laser wavelength, but differed in the power (2–2.5 W) and operating mode—pulse or continuous. Different results in terms of BOP, GI (Gingival Index), Pl.I (Plaque index) OHI (Oral Hygiene Index) in the 980 nm laser study were obtained by Zare et al. [20] and Fenol et al. [39]. In both of these studies, single laser exposure was used in continuous wave mode with 0.84 W [39] and 1 W [17]. In a 2-month observation period, they obtained significantly better improvement in BOP [17] and GI and OHI [39], than compared with their control groups, where only SRP was used. In our study, the lack of any significant difference in PCR reduction compared with the non-laser group meant that laser therapy does not prevent biofilm deposition on an exposed tooth. Also, triple irradiation with a 980 nm 1 W laser had no effect on the BOP indicator, which is directly related to inflammation and associated with an increased risk of periodontitis progression.In our study, a 980 nm diode laser turned out to be effective in reducing periodontal pockets ≥ 7 mm. In medium-deep pockets (4–6 mm), the additional use of the laser did not offer any benefits compared with the SRP itself. This result contradicts the study by Dukić et al. [30], who obtained a greater reduction in 4–6 mm pockets with 980 nm, 2 W pulsed (25 ms pulse interval) DL, though in deeper 7–10 mm pockets this effect was not noted. A positive effect on the reduction of the PPD was also demonstrated by Kamma et al. [33] (laser settings: 980 nm, 2 W, CW, power density: 2830 W/cm^2^, fluence: 94.3 J/ cm^2^, 30 s of laser application per pocket) and Fenol et al. [39] (laser settings: 980 nm, 0.84 W, CW, 0.8 J/s, duration of laser application not mentioned), while Caruso et al. [34] (laser settings: 980 nm, 2.5 W, 30 Hz, 10 ms pulse duration, 30 s of laser application with 60 s pause) and Yadwad et al. [38] (laser settings: 980 nm, 2.0 W, CW, 30 s of laser application per pocket twice with 60 s pause) did not observe differences between groups. It is believed that the reduction in pocket depth is due to the deepitalization of the pockets [35]. In our study, DL had no effect on the level of connective tissue attachment. No additional effect on CAL was also observed in studies of Dukić et al. [30], Caruso et al. [34], Yadwad et al. [38], while Kamma et al. [33] and Fenol et al. [39] observed periodontal clinical attachment level improvement after single laser irradiation. The question remains whether an accumulation of dose in repeated exposure does not inhibit any regeneration of connective tissue attachment.

Within our study, total bacterial count was significantly reduced at 3-month observation in test group. Initially, reduction after treatment (E2) was found to be close in both groups. Bacterial reduction remained stable in the laser group, while in the control group the TBC returned to its original level in E3 (Fig. 4). A greater reduction of *E. n.* was observed in the laser group. The number of bacteria *P. g.*, *T. f.*, *T. d.* and *M. m.* was also statistically significantly reduced, but this took place in both groups. Laser use did not affect the amount of *A. a.*, *P. i.*, *F. n.* and *C. g.* In the control group, on the other hand, the *P. i.* level was significantly reduced and the level of *C. g.* increased.

No additional effect of DL in test group (SRP + DL) compared with control group (SRP only) on bacterial reduction was observed by Caruso et al. [34] and by Yawad et al. [38] in split mouth designed study (with 13 participants) and in parallel study (with 30 individuals in each group) respectively. Though, Fenol et al. [39] described a statistically significant reduction in *P. g.*, *T. f.* and *T. d.* in test group (SRP + DL) comparing with control group (SRP alone) after one laser irradiation with 0.84 W with a 2-month observation period in split mouth designed study with 20 participants in. Also Gojkov-Vukelic et al. [40] described significant *P. g.* and *A. a.* reduction in SRP + DL group (laser settings: 980 nm, 2 W, 25 Hz, laser exposure: 25 s per pocket repeated twice in 5 days interval). Additionally, Kamma et al. [33] also described significant *P. g.*, *T. d.* and total bacterial load reduction in laser group after a 6 month observation period in a split mouth designed study with 30 participants. For other diode laser radiation lengths, the results of additional use of DL after SRP were also inconclusive. 805–810 nm lasers are characterized by high absorption in haemoglobin, which may be associated with an increased risk of thermal damage if the root surface is covered with blood. Hence, the recommendations to irradiate the pockets at another appointment (in few days)or rinse the pocket with saline before irradiation to remove blood from the pocket [17]. The potentially harmful effects of the 808 nm laser on the periodontium could might have been demonstrated by the results of De Micheli et al. [22] (laser settings: 808 nm, 1.5 W, CW, power density: 1193.7 W/cm^2^, irradiation time: 20 s per pocket) who obtained worse CAL and PPD results in the laser group than in the non-laser group. In the same study, both groups did not differ in terms of Pl.I. and BOP indicators and total bacteria load and *P. g.*, *A. a.*, *P. i.* levels. No antibacterial effect was observed by Euzebio Alves et al. [31] (laser settings: 808 nm, 1.5 W, CW, power density: 1193.7 W/cm^2^, irradiation time: 20 s per pocket, repeated twice in 1-week interval). Their findings are in contrast to the observations of Moritz et al. [41] (laser settings: 805 nm, 2.5 W, 50 Hz, 10 ms pulse duration, the pocket depth in mm corresponded to the exposure time in seconds), Bansal et al. [42] (laser settings: 808 nm, 0.4 W, CW with 20 s exposure per site and 0.8 W pulsed mode with 10 s exposure per tooth site) and Giannelli et al. [43] (laser settings: 810 nm, 1 W, CW, power density: 353.4 W/cm^2^, fluence: 66.7 J/cm^2^) where there was a significant reduction of periopathogens in the laser therapy group. In addition, Giannelli et al. [43] showed that a 810 nm diode laser caused the eradication of *P. g.*, *A. a*., *F. n*., *T. d.*, *P. i*. and *E. c.* extracellularly and intracellularly without damaging connective tissue and blood vessels. Kreisler et al. [35], Moritz et al. [41] and Giannelli et al. [43] saw the positive effect on PPD in the laser group. Moritz et al. [41] also noted BOP improvement in their laser group. In contrast, Euzebio Alves et al. [31] and Kreisler et al. [35] (laser settings: 809 nm, 1 W, CW, exposure time: 10 s per pocket) did not observe differences between the groups in this respect. Improvement in CAL was observed by Kreisler et al. [35] in laser group while De Micheli et al. [22] noted a deterioration. In turn, Euzebio Alves et al. [31] and Lin et al. [23] (laser settings: 810 nm, 2 W, CW, treatment time: single-rooted teeth: 1. 66 ± 0.25 s, multi-rooted teeth: 2.88 ± 0.27 s) did not observe differences between the groups. These aforementioned studies generally referred to healthy patients. There are also studies showing that the additional use of a diode laser in individuals with type 2 diabetes and periodontitis could offer measurable benefits. Chandra and Shashikumar [44] (laser settings: 808 nm, 1.5–1.8 W, CW, the pocket depth in mm corresponded to the exposure time in seconds) and Koçak et al. [45] (laser settings: 940 nm, 1.5 W, pulsed mode: 20 ms on, 20 ms off, 20 J/cm^2^, exposure time: 20 s per tooth) noted a better improvement in clinical parameters and HbA1c levels in a group of patients who received additional diode laser therapy compared with patients treated with SRP only.

The limitations in our study were patients might become aware of the group they were assigned to, the short 3-month observation period, a combination of single- and multi-rooted teeth in the analysis, the multitude of comorbidities and medications used in patients participating in the study.

## Conclusions

1. Additional use of the 980 nm, 1 W, CW laser enables a significant reduction in pocket depth ≥ 7 mm (*p* = 0.0151), which allows the patient to avoid surgical treatment after myocardial infarction; no effect was seen in shallower pockets.
2. Diode laser reduces total bacteria count and delays recolonisation during a 3-month observation period (*p* = 0.0154).
3. Additional use of a diode laser after SRP has no significant effect on BOP, CAL and PCR.
4. Additional use of a diode laser in patients with periodontitis and after myocardial infarction does not further reduce the levels of *Porphyromonas gingivalis*, *Treponema denticola* and *Tannerella forsythia*, which are key pathogens connecting periodontitis and CVD.
5. A significant increase in the number of *Capnocytophaga gingivalis* bacteria was observed in the control group (*p* = 0.048).

Within the limitations of our study, we can conclude that 980 nm diode laser can be a useful tool in the treatment of periodontitis in patients after myocardial infarction.
